# IL-33 Augments Virus-Specific Memory T Cell Inflation and Potentiates the Efficacy of an Attenuated Cytomegalovirus-Based Vaccine

**DOI:** 10.4049/jimmunol.1701757

**Published:** 2019-01-13

**Authors:** James E. McLaren, Mathew Clement, Morgan Marsden, Kelly L. Miners, Sian Llewellyn-Lacey, Emma J. Grant, Anzelika Rubina, Silvia Gimeno Brias, Emma Gostick, Maria A. Stacey, Selinda J. Orr, Richard J. Stanton, Kristin Ladell, David A. Price, Ian R. Humphreys

**Affiliations:** * Division of Infection and Immunity, Cardiff University School of Medicine, Cardiff CF14 4XN, United Kingdom;; † Infection and Immunity Program, Department of Biochemistry and Molecular Biology, Biomedicine Discovery Institute, Monash University, Clayton, Victoria 3800, Australia; and; ‡ Wellcome Trust Sanger Institute, Hinxton, Cambridge CB10 1SA, United Kingdom

## Abstract

Candidate vaccines designed to generate T cell–based immunity are typically vectored by nonpersistent viruses, which largely fail to elicit durable effector memory T cell responses. This limitation can be overcome using recombinant strains of CMV. Proof-of-principle studies have demonstrated the potential benefits of this approach, most notably in the SIV model, but safety concerns require the development of nonreplicating alternatives with comparable immunogenicity. In this study, we show that IL-33 promotes the accumulation and recall kinetics of circulating and tissue-resident memory T cells in mice infected with murine CMV. Using a replication-deficient murine CMV vector, we further show that exogenous IL-33 boosts vaccine-induced memory T cell responses, which protect against subsequent heterologous viral challenge. These data suggest that IL-33 could serve as a useful adjuvant to improve the efficacy of vaccines based on attenuated derivatives of CMV.

## Introduction

Recombinant viruses have been widely explored as vaccine vectors purposed to elicit T cell immunity (1–3). In immunocompetent hosts, wild-type (WT) human CMV (HCMV) typically establishes an asymptomatic, lifelong infection. Unlike many other viruses, however, HCMV induces unusually large T cell responses that expand over time, a process termed “memory inflation” (4, 5). The majority of circulating HCMV-specific CD4^+^ and CD8^+^ T cells display highly differentiated phenotypes associated with the acquisition and rapid deployment of antiviral effector functions (6, 7). Moreover, in vivo studies have shown that murine CMV (MCMV) drives the formation of tissue-resident memory T (T_RM_) cells (8–10). Coupled with the ability of different strains to “superinfect” seropositive individuals (11), these data prompted speculation that HCMV may serve as a unique vector, enabling the generation of local and systemic T cell immunity against heterologous Ags (12). This approach was subsequently validated with astonishing results in the SIV model, where vaccine-elicited effector memory T (T_EM_) cells protected rhesus macaques from viral challenge and cleared established infections (13–15). Similarly, replication-competent strains of MCMV have been used as vectors to protect mice from Ebola virus (16), HSV type 1 (17), and various cancers (18–20). However, this strategy is limited in humans, where replication-competent vaccines pose serious risks to immunocompromised recipients and hold the potential to recombine unpredictably with naturally occurring strains of HCMV. Accordingly, much effort has focused on the development of replication-deficient alternatives (21–24). Of particular note, glycoprotein L–deficient (ΔgL) MCMV vectors have been shown to elicit sustained CD8^+^ T_EM_ cell responses (22–24), with demonstrable effects in murine cancer models (25). These vectors nonetheless deliver a modest antigenic stimulus compared with replication-competent strains of MCMV (22–24).

IL-33, a member of the IL-1 cytokine family, is released as a danger signal or “alarmin” in response to infection or cellular stress (26, 27) and exhibits a wide range of functions that aid immune clearance of microbes and parasites (28). Although IL-33 can act in the nucleus of healthy nonhematopoietic cells, it signals as an alarmin via ST2 and the IL-1R accessory protein, which combine to form an active heterodimeric receptor on the surface of macrophages, NK cells, and T cells (28). Divergent immunological effects have been ascribed to IL-33. In some settings, it induces regulatory T cell expansions (28–30), whereas in other settings, it promotes antiviral T cell immunity (31–33). IL-33 can also enhance the production of virus-specific Abs at mucosal surfaces and boost the immunogenicity of DNA and protein-based vaccines (31, 34–36). It is further notable that alum, a long-established vaccine adjuvant, induces the release of IL-33 (37).

In this study, we show that IL-33 augments memory T cell inflation and recall, as well as the formation of classically defined (CD69^+^) T_RM_ cells, in mice infected with MCMV. We also demonstrate that IL-33 enhances replication-deficient (ΔgL) MCMV vaccine-induced memory T cell responses, leading to greater protection against subsequent heterologous viral challenge. Collectively, these data suggest that the translational benefits of attenuated CMV-based vaccines can be potentiated by alarmins, such as IL-33.

## Materials and Methods

### Mice, infections, and treatments

*Il33^−/−^* mice were bred in-house (38). C57BL/6 mice were purchased from Charles River Laboratories or Envigo. Sex-matched mice aged 7–9 wk were used in all experiments. Smith-strain MCMV was propagated in vivo and prepared via sorbitol gradient purification (39). Mice were infected i.p. with 3 × 10^4^ PFU of MCMV. ΔgL-SL8-MCMV was prepared as described previously (24). Mice were infected i.p. with 4 × 10^5^ PFU of ΔgL-SL8-MCMV. In some experiments, 2 μg of rIL-33 (BioLegend) was administered i.p. at the time of infection. Replication-deficient recombinant adenovirus type 5 (pAdZ5-CV5) expressing immediate-early protein 3 (rAd-IE3) was engineered and purified as described previously (40). Mice were challenged i.p. with 5 × 10^8^ PFU of rAd-IE3 or 2 × 10^6^ PFU of recombinant vaccinia virus expressing OVA (rVV-OVA) (41). All mouse experiments were performed at Cardiff University under U.K. Home Office Project License 30/2969 (London, U.K.).

### Tetramers

Fluorochrome-tagged H-2D^b^/M45 (HGIRNASFI), H-2K^b^/m139 (TVYGFCLL), H-2K^b^/M38 (SSPPMFRV), H-2K^b^/IE3 (RALEYKNL), and H-2K^b^/SL8 (SIINFEKL) tetramers were produced in-house as described previously (42).

### Flow cytometry

Leukocytes were isolated from spleens, lungs, salivary glands, and inguinal lymph nodes (LNs) as described previously (43). Peripheral blood was collected from the lateral tail vein directly into heparin-coated tubes via an integrated capillary (Sarstedt).

For phenotypic analyses of CD4^+^ and CD8^+^ T cells, leukocytes were stained with LIVE/DEAD Fixable Aqua (Thermo Fisher Scientific), incubated with anti-CD16/CD32 Fc-block (BioLegend), and stained with various combinations of the following mAbs: anti-CD62L–PE-Texas Red (clone MEL-14; BD Biosciences); anti-CD69–PE-Cy7 (clone H1.2F3; eBioscience); anti-KLRG-1–FITC (clone 2F1; Southern Biotech); and anti-CD3ε–PE-Cy5 (clone 145-2C11), anti-CD4–allophycocyanin-Fire 750 (clone RM4-5), anti-CD8α–BV711 (clone 53-6.7), anti-CD11a–allophycocyanin (clone M17/4), anti-CD27–allophycocyanin (clone LG.3A10), anti-CD44–PE-Cy7 (clone IM7), anti-CD62L–PE-Dazzle 594 (clone MEL-14), anti-CD69–allophycocyanin (clone H1.2F3), anti-CD103–PE (clone 2E7), anti-CD103–PE-Dazzle 594 (clone 2E7), and anti-PD-1–BV605 (clone 29F.1A12) (all from BioLegend). Ag-specific CD8^+^ T cells were prelabeled with tetramer as indicated for 20 min at 37°C (44).

The following mAbs were used to characterize dendritic cells (DCs), myeloid cells, and NK cells: anti-I-A/I-E–Pacific Blue (clone M5/114.15.2), anti-CD11b–FITC (clone M1/70), anti-CD11c–PE (clone N418), anti-CD40–FITC (clone 3/23), anti-CD45R/B220–BV785 (clone RA3-6B2), anti-CD80–allophycocyanin (clone 16-0A1), anti-CD86–PE-Cy7 (clone GL-1), anti-F480–BV711 (clone BM8), anti-Ly6C–PE-Cy7 (clone HK1.4), anti-Ly6G–PE-Dazzle 594 (clone 1A8), anti-Ly49H–PE-Cy7 (clone 3D10), anti-NK1.1–BV421 (clone PK136), and anti-SiglecH–allophycocyanin (clone 551) (all from BioLegend).

For functional analyses of CD8^+^ T cells, leukocytes were stimulated for 6 h with the indicated peptide (M45, m139, M38, IE3, or SL8) at a final concentration of 2 μg/ml in the presence of monensin (1:1500 stock dilution; BD Biosciences), brefeldin A (2 μg/ml; BD Biosciences), and anti-CD107a–FITC (clone 1D4B; BioLegend). Cells were then stained with LIVE/DEAD Fixable Aqua (Thermo Fisher Scientific), incubated with anti-CD16/CD32 Fc-block (BioLegend), and stained with anti-CD4–allophycocyanin-Fire 750 (clone RM4-5) and anti-CD8α–BV711 (clone 53-6.7) (both from BioLegend). After fixation/permeabilization using a standard paraformaldehyde/saponin protocol, cells were further stained intracellularly with anti-IFN-γ–Pacific Blue (clone XMG1.2) and anti-TNF-α–PE-Cy7 (clone MP6-XT22) (both from BioLegend).

Intravascular staining was performed using anti-CD8α–PE-Cy5 (clone 53-6.7; BioLegend) at a dose of 3 μg/mouse as described previously (8, 9, 45). All data were acquired using a modified FACSAria II flow cytometer (BD Biosciences) and analyzed with FlowJo software version 9.9.3 (Tree Star).

### Real-time quantitative PCR

Total RNA was extracted from cell populations and whole tissues using an SV Total RNA Isolation Kit (Promega). cDNA was synthesized using Superscript II (Thermo Fisher Scientific). Expression of *Il33* was quantified using a MiniOpticon Real-Time PCR System (Bio-Rad Laboratories) with Platinum SYBR Green Master Mix (Bio-Rad Laboratories). Specific primers were described previously (43, 46). Expression of sphingosine-1-phosphate receptor 1 (*S1pr1*) was quantified using a QuantStudio 3 Real-Time PCR System (Applied Biosystems) with a TaqMan Gene Expression Assay (Mm02619656_s1) and TaqMan Fast Advanced Master Mix (Applied Biosystems). All data were normalized to β-actin (*ACTB*).

### Quantification of viral DNA

Saliva was obtained from anesthetized mice via oral lavage of the sublingual cavity using 20 μl of PBS (47). Viral DNA was quantified in 1 μl of sample using an *Ie1*-specific quantitative PCR. DNA copies per microliter were determined against a standard curve established using an MCMV pARK25 bacterial artificial chromosome (a kind gift from A. Redwood, Murdoch University, Perth, Australia).

### Statistical analysis

Data were evaluated for statistical significance using the Mann–Whitney *U* test (two groups) or a one-way ANOVA followed by the Tukey post hoc test (>2 groups) in Prism Mac 5.0f (GraphPad). Virus load data were logarithmically transformed prior to analysis. Outliers were included in all datasets. All *p* values are reported as follows: *p ≤ 0.05, **p ≤ 0.01, and ***p ≤ 0.001.

## Results

### IL-33 is induced in lymphoid organs during acute MCMV infection but is not required for early innate responses or the induction of virus-specific CD8^+^ T cells

**FIGURE 1.**
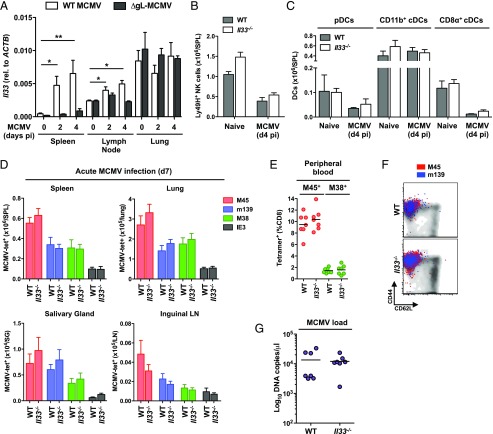
IL-33 is induced in lymphoid organs during acute MCMV infection but is not required for early innate responses or the induction of virus-specific CD8^+^ T cells. (**A**) C57BL/6 mice were infected with MCMV (WT) or ΔgL-SL8-MCMV. Spleens, lungs, and inguinal LNs were isolated on day 2 or day 4 postinfection (pi). Naive mice were used as the day 0 control. Expression of *Il33* was measured using a real-time quantitative PCR. Absolute values were normalized to *ACTB*. Data are shown as mean ± SEM (*n* = 4 mice per group). *p ≤ 0.05, **p ≤ 0.01. (**B**–**F**) C57BL/6 (WT) and *Il33^−/−^* mice were infected with MCMV. (B) Total numbers of Ly49H^+^ NK cells (CD3^–^NK.1.1^+^) were quantified among leukocytes isolated from spleens on day 4 pi. Data are shown as mean ± SEM (*n* = 5 mice per group). (C) Total numbers of DCs (CD3^–^CD11c^+^MHCII^+^) were quantified among leukocytes isolated from spleens on day 4 pi as follows: plasmacytoid DCs (pDCs) (B220^+^CD11b^–^Ly6C^+^SigLec-H^+^), CD11b^+^ cDCs (B220^–^CD8α^–^CD11b^+^), and CD8α^+^ cDCs (B220^–^CD8α^+^CD11b^–^). Data are shown as mean ± SEM (*n* = 5 mice per group). (D) Total numbers of tetramer-binding CD8^+^ T cells specific for M45, m139, M38, or IE3 were quantified among leukocytes isolated from spleens, lungs, salivary glands, and inguinal LNs on day 7 pi. Data are shown as mean ± SEM (*n* = 5 mice per group). (E) Frequencies of tetramer-binding CD8^+^ T cells specific for M45 or M38 were quantified in peripheral blood on day 7 pi. Horizontal bars depict mean percentage of viable CD3^+^CD8^+^ T cells (*n* = 7 mice per group). (F) Expression levels of CD44 and CD62L were quantified on tetramer-binding CD8^+^ T cells specific for M45 or m139 among leukocytes isolated from spleens on day 7 pi. Representative flow cytometry plots are shown. Ag-specific events are depicted as colored dots superimposed on black density plots encompassing all CD8^+^ T cells. (**G**) Viral genomes were quantified in saliva via quantitative PCR on day 14 pi. Horizontal bars depict median log_10_ DNA copies per microliter (*n* = 7 mice per group). All results are shown (A–E and G).

MCMV initially targets stromal cells in the marginal zone of the spleen (48), which express high levels of IL-33 (26). Other viruses, such as lymphocytic choriomeningitis virus (LCMV), target fibroblastic reticular cells (49) and enhance *Il33* gene expression (31). We found that *Il33* was upregulated in the spleen and inguinal LNs, but not the lung, during acute MCMV infection (Fig. 1A). Alongside previously reported data (50, 51), these results suggest that MCMV induces *Il33* expression in fibroblastic reticular cells, which are not present in mucosal tissues (52). In contrast, the replication-deficient strain ΔgL-MCMV did not significantly upregulate *Il33* (Fig. 1A).

IL-33 can influence the development and function of NK cells (50) and APCs (28, 53, 54), which act synergistically to provide acute-phase antiviral immunity (55, 56). To investigate these effects in the context of MCMV, we infected WT and *Il33^−/−^* mice (38) and measured the accumulation of Ly49H^+^ NK cells (Fig. 1B), which limit viral replication in this model (57), B cells (Supplemental Fig. 1A), and various subsets of DCs, including conventional DCs (cDCs) and plasmacytoid DCs (Fig. 1C). No clear differences in absolute cell numbers were observed between WT and *Il33^−/−^* mice. Moreover, Ly49H^+^ NK cells were activated to a similar extent in WT and *Il33^−/−^* mice (Supplemental Fig. 1B), and pairwise comparisons of CD11b^+^ and CD8α^+^ cDCs across strains revealed comparable expression levels of MHC class II and the costimulatory molecules CD40, CD80, and CD86 (Supplemental Fig. 1C). These findings suggest that MCMV triggers innate responses independently of IL-33.

IL-33 promotes antiviral CD8^+^ T cell immunity during acute LCMV infection (31). To extend this observation, we characterized MCMV-specific CD8^+^ T cell responses in WT and *Il33^−/−^* mice using MHC class I–peptide tetramers corresponding to the noninflationary H-2D^b^–restricted M45 epitope and the inflationary H-2K^b^–restricted m139, M38, and IE3 epitopes (58). On day 7 postinfection, similar numbers of MCMV-specific CD8^+^ T cells were detected per site in the spleens, lungs, salivary glands, and inguinal LNs of WT and *Il33^−/−^* mice (Fig. 1D). The frequencies of M45-specific and M38-specific CD8^+^ T cells in peripheral blood were also unaffected by a lack of IL-33 (Fig. 1E). In both strains of mice, all MCMV-specific CD8^+^ T cells displayed an Ag-experienced (CD44^hi^CD62L^lo^) T_EM_ phenotype, irrespective of specificity and tissue origin (Fig. 1F). Moreover, the total numbers of CD4^+^ and CD8^+^ T cells in lymphoid organs and mucosal tissues were comparable between WT and *Il33^−/−^* mice (Supplemental Fig. 1D). These data suggest that IL-33 is not required for the induction of CD8^+^ T cell immunity during acute MCMV infection.

In line with these findings, equivalent levels of viral DNA were detected in WT and *Il33^−/−^* mice on day 14 postinfection (Fig. 1G). Similar results were reported previously in a study of *St2^−/−^* BALB/c mice (51).

### Endogenous IL-33 promotes MCMV-specific CD8^+^ memory T cell inflation and the accumulation of CD69^+^ T_RM_ cells

Chronic MCMV infection drives systemic CD8^+^ memory T cell inflation and the accumulation of virus-specific CD4^+^ and CD8^+^ T_RM_ cells in mucosal tissues (8–10). CD4^+^ T_RM_ cells typically express CD11a and CD69, whereas CD8^+^ T_RM_ cells typically express CD69 either alone or in conjunction with CD103 (8, 59). It has also been shown that IL-33 can upregulate the expression of CD69 on T cells, at least in vitro (9, 60). We therefore assessed the impact of IL-33 on the development of MCMV-specific T_EM_ and T_RM_ cells in vivo.

**FIGURE 2.**
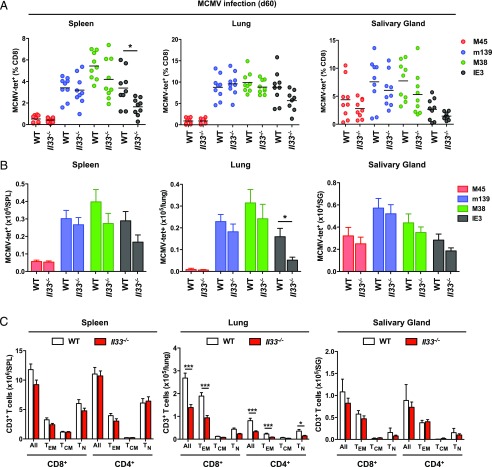
*Il33^−/−^* mice exhibit impaired virus-specific CD8^+^ memory T cell inflation during chronic MCMV infection. C57BL/6 (WT) and *Il33^−/−^* mice were infected with MCMV. (**A**) Frequencies of tetramer-binding CD8^+^ T cells specific for M45, m139, M38, or IE3 were quantified among leukocytes isolated from spleens, lungs, and salivary glands on day 60 postinfection (pi). Horizontal bars depict mean percentage of viable CD3^+^CD8^+^ T cells (*n* = 9 mice per group). (**B**) Total numbers of tetramer-binding CD8^+^ T cells specific for M45, m139, M38, or IE3 were quantified among leukocytes isolated from spleens, lungs, and salivary glands on day 60 pi. Data are shown as mean ± SEM (*n* = 12–16 mice per group). (**C**) Total numbers of naive (T_N_) and memory T (T_EM_ and T_CM_) cells within the CD4^+^ and CD8^+^ compartments were quantified among leukocytes isolated from spleens, lungs, and salivary glands on day 60 pi. Data are shown as mean ± SEM (*n* = 9–12 mice per group). Results are concatenated from three independent experiments (A–C). *p ≤ 0.05, ***p ≤ 0.001.

On day 60 postinfection, lower frequencies of IE3-specific CD8^+^ T cells were present in the spleens of *Il33*^−/−^ mice relative to WT mice, and a corresponding trend was observed in the lungs and salivary glands (Fig. 2A). Similarly, lower numbers of IE3-specific CD8^+^ T cells were present in the lungs of *Il33*^−/−^ mice relative to WT mice, and a corresponding trend was observed in the spleens and salivary glands (Fig. 2B). The total numbers of CD4^+^ and CD8^+^ T cells, especially within the T_EM_ compartment, were also lower in the lungs of *Il33*^−/−^ mice relative to WT mice (Fig. 2C). No significant differences were observed among tissues in parallel analyses of CD8^+^ T cells specific for M45, m139, or M38.

**FIGURE 3.**
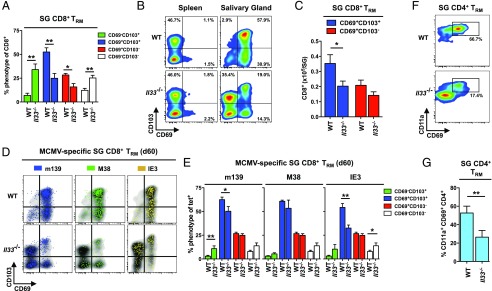
Endogenous IL-33 promotes the accumulation of CD69^+^ T_RM_ cells during chronic MCMV infection. C57BL/6 (WT) and *Il33^−/−^* mice were infected with MCMV. (**A**) Expression levels of CD69 and CD103 were quantified on CD8^+^ T cells among leukocytes isolated from salivary glands on day 60 postinfection (pi). Data are shown as mean ± SEM (*n* = 9 mice per group). (**B**) Expression levels of CD69 and CD103 were quantified on CD8^+^ T cells among leukocytes isolated from spleens and salivary glands on day 60 pi. Representative flow cytometry plots are shown. (**C**) Total numbers of CD8^+^ T_RM_ (CD69^+^CD103^–^ or CD69^+^CD103^+^) cells were quantified among leukocytes isolated from salivary glands on day 60 pi. Data are shown as mean ± SEM (*n* = 9 mice per group). (**D**) Expression levels of CD69 and CD103 were quantified on tetramer-binding CD8^+^ T cells specific for m139, M38, or IE3 among leukocytes isolated from salivary glands on day 60 pi. Representative flow cytometry plots are shown. Ag-specific events are depicted as colored dots superimposed on black density plots encompassing all CD8^+^ T cells. (**E**) Expression levels of CD69 and CD103 were quantified on tetramer-binding CD8^+^ T cells specific for m139, M38, or IE3 among leukocytes isolated from salivary glands on day 60 pi. Data are shown as mean ± SEM (*n* = 9 mice per group). (**F**) Expression levels of CD11a and CD69 were quantified on CD4^+^ T cells among leukocytes isolated from salivary glands on day 60 pi. Representative flow cytometry plots are shown. (**G**) Frequencies of CD4^+^ T cells expressing CD11a and CD69 were quantified among leukocytes isolated from salivary glands on day 60 pi. Data are shown as mean ± SEM (*n* = 9–12 mice per group). Results are concatenated from three independent experiments (A, C, E, and G). *p ≤ 0.05, **p ≤ 0.01.

In the spleens and lungs of WT and *Il33^−/−^* mice chronically infected with MCMV, the vast majority of CD8^+^ T cells were CD69^–^CD103^–^ or CD69^–^CD103^+^ (Supplemental Fig. 2A). Virus-specific inflationary CD8^+^ T cells in these organs were also predominantly CD69^–^CD103^–^, as reported previously (8, 9), with no discernible phenotypic differences between WT and *Il33^−/−^* mice (Supplemental Fig. 2B). However, in the salivary glands, where CD69^+^ T_RM_ cells accumulate during chronic MCMV infection (8, 9), the frequencies (Fig. 3A, 3B) and total numbers (Fig. 3C) of CD8^+^ T_RM_ (CD69^+^CD103^–^ or CD69^+^CD103^+^) cells were greatly reduced in *Il33*^−/−^ mice. The frequencies of inflationary MCMV-specific CD8^+^ T_RM_ cells with a CD69^+^CD103^+^ phenotype, especially those specific for IE3, were also reduced in *Il33^−/−^* mice (Fig. 3D, 3E), although no significant differences were observed with respect to overall numbers (Supplemental Fig. 2C). Similarly, the frequencies of CD4^+^ T_RM_ (CD11a^+^CD69^+^) cells were lower in the salivary glands of *Il33*^−/−^ mice relative to WT mice (Fig. 3F, 3G).

Collectively, these data identify IL-33 as an important mediator of virus-specific CD8^+^ memory T cell inflation and CD69^+^ T_RM_ cell formation during chronic MCMV infection.

### IL-33–driven upregulation of CD69 is not required for the retention of CD8^+^ T_RM_ cells during chronic MCMV infection

CD69 is commonly used as a marker to distinguish bona fide T_RM_ cells from recirculating T_EM_ cells (61), but tissue residency per se is not defined by the expression of CD69 (62). We therefore used intravascular staining to determine if *Il33*^−/−^ mice truly lack the ability to generate CD8^+^ T_RM_ cells after infection with MCMV. In WT mice, tissue-localized CD8^+^ T cells predominated in the salivary glands, as reported previously (8, 9), whereas intravascular CD8^+^ T cells predominated in the lungs (Supplemental Fig. 2D, 2E). A similar pattern was observed in *Il33*^−/−^ mice chronically infected with MCMV (Supplemental Fig. 2D, 2E). Moreover, comparable frequencies of CD69^–^CD103^–^ and CD69^–^CD103^+^ CD8^+^ T cells were retained in the salivary glands of WT and *Il33*^−/−^ mice (Supplemental Fig. 2F).

Tissue egress is mediated by S1PR1, which is downregulated by CD69 (63). To examine the role of IL-33 in this process, we measured *S1pr1* expression in flow-purified CD69^–^CD103^+^ and CD69^+^CD103^+^ CD8^+^ T cells isolated from the salivary glands of WT and *Il33*^–^*^/^*^–^ mice chronically infected with MCMV. In both strains of mice, CD69^–^CD103^+^ CD8^+^ T cells expressed higher levels of *S1pr1* than CD69^+^CD103^+^ CD8^+^ T cells, although these differences lacked significance in pairwise comparisons (Supplemental Fig. 2G). Moreover, CD69^–^CD103^+^ CD8^+^ T cells expressed higher levels of *S1pr1* in *Il33*^–^*^/^*^–^ mice relative to WT mice, albeit just below the threshold for significance (Supplemental Fig. 2G).

Collectively, these data suggest that IL-33 is not mandatory for the retention of CD8^+^ T_RM_ cells during chronic MCMV infection, potentially because a subsidiary mechanism fulfills this role independently of CD69 (64).

### Exogenous IL-33 enhances MCMV-specific CD8^+^ memory T cell inflation and the accumulation of CD69^+^ T_RM_ cells

**FIGURE 4.**
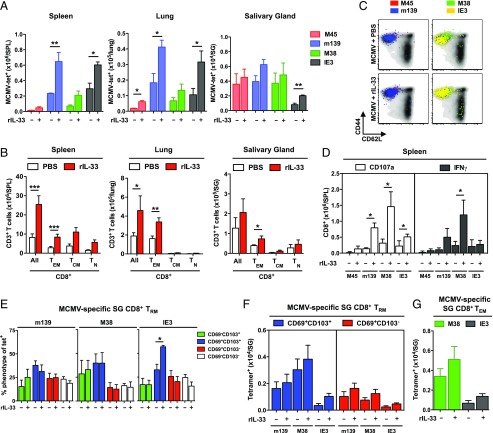
Exogenous IL-33 enhances virus-specific memory T cell inflation and the accumulation of CD69^+^ T_RM_ cells during chronic MCMV infection. C57BL/6 mice were infected with MCMV alongside a single dose of rIL-33 (2 μg) or volume-equivalent PBS. (**A**) Total numbers of tetramer-binding CD8^+^ T cells specific for M45, m139, M38, or IE3 were quantified among leukocytes isolated from spleens, lungs, and salivary glands on day 60 postinfection (pi). Data are shown as mean ± SEM (*n* = 5–9 mice per group). (**B**) Total numbers of naive (T_N_) and memory T (T_EM_ and T_CM_) cells within the CD8^+^ compartment were quantified among leukocytes isolated from spleens, lungs, and salivary glands on day 60 pi. Data are shown as mean ± SEM (*n* = 5–9 mice per group). (**C**) Expression levels of CD44 and CD62L were quantified on tetramer-binding CD8^+^ T cells specific for M45, m139, M38, or IE3 among leukocytes isolated from spleens on day 60 pi. Representative flow cytometry plots are shown. Ag-specific events are depicted as colored dots superimposed on black density plots encompassing all CD8^+^ T cells. (**D**) Total numbers of functional (CD107a^+^ or IFN-γ^+^) CD8^+^ T cells specific for M45, m139, M38, or IE3 were quantified among leukocytes isolated from spleens on day 60 pi. Data are shown as mean ± SEM (*n* = 4 mice per group). (**E**) Expression levels of CD69 and CD103 were quantified on tetramer-binding CD8^+^ T cells specific for M45, m139, M38, or IE3 among leukocytes isolated from salivary glands on day 60 pi. Data are shown as mean ± SEM (*n* = 5 mice per group). (**F**) Total numbers of tetramer-binding CD8^+^ T_RM_ (CD69^+^CD103^–^ or CD69^+^CD103^+^) cells specific for m139, M38, or IE3 were quantified among leukocytes isolated from salivary glands on day 60 pi. Data are shown as mean ± SEM (*n* = 5 mice per group). (**G**) Total numbers of tetramer-binding CD8^+^ T_EM_ (CD44^hi^CD62L^lo^) cells specific for M38 or IE3 were quantified among leukocytes isolated from salivary glands on day 60 pi. Data are shown as mean ± SEM (*n* = 5 mice per group). Results are concatenated from two independent experiments (A, B, and D–G). *p ≤ 0.05, **p ≤ 0.01, ***p ≤ 0.001.

Early immunological signals can modulate the development of MCMV-specific memory T cells (65–67). On this basis, we administered a single dose (2 μg) of rIL-33 at the time of MCMV infection and enumerated virus-specific CD8^+^ T cells in WT mice. In line with our studies of *Il33^−/−^* mice (Fig. 1D–F), rIL-33 did not influence virus-specific CD8^+^ T cell responses in the spleen during acute MCMV infection (Supplemental Fig. 3A, 3B). On day 7 postinfection, however, greater numbers of IE3-specific CD8^+^ T cells were present in the inguinal LNs of mice treated with rIL-33 (Supplemental Fig. 3C). A similar pattern was observed across naive and memory subsets of CD8^+^ T cells (Supplemental Fig. 3D). Moreover, rIL-33 greatly enhanced the numbers of inflationary MCMV-specific (Fig. 4A) and total (Fig. 4B) CD8^+^ T cells in the spleen during chronic MCMV infection (day 60 postinfection), all of which retained a predominant CD44^hi^CD62L^lo^ phenotype (Fig. 4C). A corresponding increase in the numbers of functional (CD107a^+^ and/or IFN-γ^+^) inflationary MCMV-specific CD8^+^ T cells was also detected in mice treated with rIL-33 (Fig. 4D). In addition, rIL-33 enhanced the numbers of inflationary MCMV-specific CD8^+^ T cells in mucosal tissues (Fig. 4A), alongside a generic increase in the accumulation of total CD8^+^ T cells, especially within the T_EM_ compartment (Fig. 4B).

Endogenous IL-33 promotes the accumulation of CD69^+^ T_RM_ cells in the salivary glands during chronic MCMV infection (Fig. 3). In line with this finding, rIL-33 enhanced the frequencies of IE3-specific CD8^+^ T_RM_ (CD69^+^CD103^+^) cells (Fig. 4E), although corresponding increases in the numbers of inflationary MCMV-specific CD8^+^ T_RM_ and T_EM_ cells failed to achieve significance (Fig. 4F, 4G).

Collectively, these data suggest that rIL-33 can influence Ag-driven priming events during acute MCMV infection to enhance virus-specific CD8^+^ memory T cell inflation and the accumulation of CD69^+^ T_RM_ cells during chronic MCMV infection.

### Exogenous IL-33 augments the immunogenicity and efficacy of a model ΔgL-MCMV–based vaccine

**FIGURE 5.**
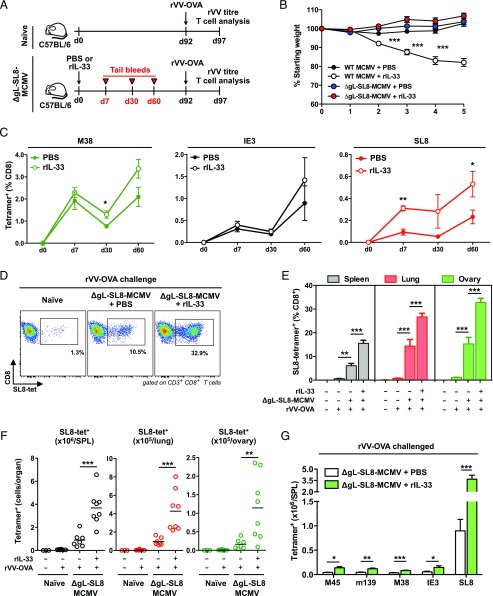
Exogenous IL-33 boosts the immunogenicity of ΔgL-SL8-MCMV. Female C57BL/6 mice were infected with ΔgL-SL8-MCMV alongside a single dose of rIL-33 (2 μg) or volume-equivalent PBS. (**A**) Schematic representation of the experimental protocol. Peripheral blood was drawn on day 7, day 30, and day 60 postinfection (pi). Mice were challenged with rVV-OVA on day 92 pi. Spleens, lungs, and ovaries were harvested on day 97 pi. Naive mice were challenged in parallel as controls. (**B**) Body weight was measured in each group of ΔgL-SL8-MCMV–infected mice and the corresponding groups of WT MCMV-infected mice. Data are shown as mean ± SEM (*n* = 7 mice per group). (**C**) Frequencies of tetramer-binding CD8^+^ T cells specific for M38, IE3, or SL8 were quantified in peripheral blood on day 7, day 30, and day 60 pi. Data are shown as mean ± SEM (*n* = 3–8 mice per group). (**D**) Frequencies of tetramer-binding CD8^+^ T cells specific for SL8 were quantified among leukocytes isolated from ovaries on day 5 postchallenge (day 97 pi). Representative flow cytometry plots are shown. (**E**) Frequencies of tetramer-binding CD8^+^ T cells specific for SL8 were quantified among leukocytes isolated from spleens, lungs, and ovaries on day 5 postchallenge (day 97 pi). Data are shown as mean ± SEM (*n* = 3–8 mice per group). (**F**) Total numbers of tetramer-binding CD8^+^ T cells specific for SL8 were quantified among leukocytes isolated from spleens, lungs, and ovaries on day 5 postchallenge (day 97 pi). Data are shown as mean ± SEM (*n* = 3–8 mice per group). (**G**) Total numbers of tetramer-binding CD8^+^ T cells specific for M45, m139, M38, IE3, or SL8 were quantified among leukocytes isolated from spleens on day 5 postchallenge (day 97 pi). Data are shown as mean ± SEM (*n* = 3–8 mice per group). Results are concatenated from two independent experiments (B, C, and E–G). *p ≤ 0.05, **p ≤ 0.01, ***p ≤ 0.001.

Replication-competent CMV vectors are highly potent immunogens, delivering remarkable efficacy as preventive and therapeutic vaccines in multiple settings (10–20). However, safety concerns mandate the development of nonreplicating alternatives for widespread use in human populations. Although the replication-deficient strain ΔgL-MCMV drives virus-specific memory T cell inflation in mice, it remains substantially less immunogenic than replication-competent strains of MCMV (22–24). In light of our results with rIL-33, we hypothesized that similar effects could be harnessed to augment the immunogenicity and efficacy of a model ΔgL-MCMV–based vaccine, especially as viral replication is required to induce endogenous IL-33 expression in the spleens and inguinal LNs of acutely infected mice (Fig. 1A). To test this idea, we immunized mice with a recombinant ΔgL-MCMV expressing the H2-K^b^–restricted SIINFEKL (SL8) epitope (ΔgL-SL8-MCMV) derived from OVA (24). A mock adjuvant (PBS) or a single dose of rIL-33 (2 μg) was administered in parallel at the time of infection (Fig. 5A). After 92 d, mice were challenged with rVV-OVA (41, 68).

Mice immunized with ΔgL-SL8-MCMV plus rIL-33 exhibited no obvious weight loss during acute infection, suggesting a good adjuvant safety profile with no obvious toxicity, in contrast to mice immunized with WT MCMV plus rIL-33 (Fig. 5B). The vector itself elicited higher frequencies of circulating CD8^+^ T cells specific for M38 and IE3 in mice immunized with ΔgL-SL8-MCMV plus rIL-33 relative to mice immunized with ΔgL-SL8-MCMV plus PBS, although these differences were modest and largely fell below the threshold for significance (Fig. 5C). The frequencies of circulating SL8-specific CD8^+^ T cells, most of which displayed a T_EM_ phenotype (Supplemental Fig. 4A), also increased over time to a greater extent in the ΔgL-SL8-MCMV plus rIL-33 group compared with the ΔgL-SL8-MCMV plus PBS group (Fig. 5C). Encouragingly, these insert-specific differences were significant at early (day 7) and late (day 60) time points after immunization with ΔgL-SL8-MCMV. In addition, the circulating frequencies of total CD8^+^ T_EM_ cells were initially higher in mice immunized with ΔgL-SL8-MCMV plus rIL-33 relative to mice immunized with ΔgL-SL8-MCMV plus PBS (Supplemental Fig. 4B).

In response to rVV-OVA challenge, naive mice generated nominal frequencies (Fig. 5D, 5E) and total numbers (Fig. 5F) of SL8-specific CD8^+^ T cells in the spleen, lungs, and ovaries, which are the major sites of viral replication in this model (41, 69). Substantially greater recall responses were detected in mice immunized with ΔgL-SL8-MCMV (Fig. 5D–F). Moreover, rIL-33 amplified these recall responses, such that higher frequencies (Fig. 5D, 5E) and numbers (Fig. 5F) of SL8-specific CD8^+^ T cells were present in the spleens, lungs, and ovaries of mice immunized with ΔgL-SL8-MCMV plus rIL-33 relative to mice immunized with ΔgL-SL8-MCMV plus PBS. In addition, rIL-33 substantially enhanced the total numbers of CD4^+^ and CD8^+^ T_EM_ cells in the spleens, lungs, and ovaries of mice immunized with ΔgL-SL8-MCMV (Supplemental Fig. 4C–F). To contextualize these results, a single dose of rIL-33 administered at the time of immunization with ΔgL-SL8-MCMV magnified the total SL8-specific CD8^+^ T cell response to rVV-OVA challenge by 4.1-fold in the spleen, 4.4-fold in the lungs, and 5.6-fold in the ovaries (Fig. 5F). Of note, rIL-33 also marginally enhanced the MCMV-specific CD8^+^ T cell response after challenge with rVV-OVA (Fig. 5G, Supplemental Fig. 4G).

**FIGURE 6.**
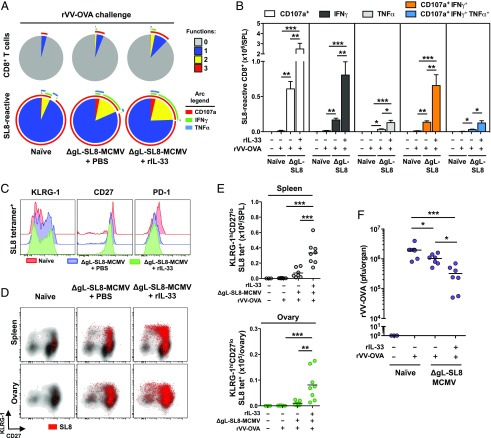
Exogenous IL-33 augments the protective efficacy of ΔgL-SL8-MCMV. Female C57BL/6 mice were infected with ΔgL-SL8-MCMV alongside a single dose of rIL-33 (2 μg) or volume-equivalent PBS and challenged with rVV-OVA on day 92 postinfection (pi) as depicted in Fig. 5A. (**A**) Functional profiles of CD8^+^ T cells specific for SL8 were characterized among leukocytes isolated from spleens on day 5 postchallenge (day 97 pi). Combination gates were exported into Simplified Presentation of Incredibly Complex Evaluations (SPICE) software (https://niaid.github.io/spice/). Pie chart segments represent the fraction of cells expressing the number of functions indicated in the key. Arcs denote individual functions (CD107, IFN-γ, and TNF-α). Concatenated data are shown (*n* = 3–8 mice per group). (**B**) Total numbers of monofunctional (CD107a^+^, IFN-γ^+^, or TNF-α^+^) and polyfunctional (CD107a^+^IFN-γ^+^ or CD107a^+^IFN-γ^+^TNF-α^+^) CD8^+^ T cells specific for SL8 were quantified among leukocytes isolated from spleens on day 5 postchallenge (day 97 pi). Data are shown as mean ± SEM (*n* = 3–8 mice per group). (**C**) Expression levels of KLRG-1, CD27, and PD-1 were quantified on tetramer-binding CD8^+^ T cells specific for SL8 among leukocytes isolated from spleens on day 5 postchallenge (day 97 pi). Representative overlay histograms are shown. (**D**) Expression levels of KLRG-1 and CD27 were quantified on tetramer-binding CD8^+^ T cells specific for SL8 among leukocytes isolated from spleens and ovaries on day 5 postchallenge (day 97 pi). Representative flow cytometry plots are shown. Ag-specific events are depicted as colored dots superimposed on black density plots encompassing all CD8^+^ T cells. (**E**) Total numbers of KLRG-1^hi^CD27^lo^ tetramer-binding CD8^+^ T cells specific for SL8 were quantified among leukocytes isolated from spleens and ovaries on day 5 postchallenge (day 97 pi). Data are shown as mean ± SEM (*n* = 3–8 mice per group). (**F**) Replicating rVV-OVA titers were quantified in ovaries isolated on day 5 postchallenge (day 97 pi). Horizontal bars depict median PFU per organ (*n* = 3–8 mice per group). Results are concatenated from two independent experiments (A, B, E, and F). *p ≤ 0.05, **p ≤ 0.01, ***p ≤ 0.001.

Immunization with ΔgL-SL8-MCMV enhanced the functional profile of SL8-specific CD8^+^ T cells responding to rVV-OVA challenge (Fig. 6A). As a consequence, greater numbers of monofunctional (CD107a^+^, IFN-γ^+^, or TNF-α^+^) and polyfunctional (CD107a^+^IFN-γ^+^ or CD107a^+^IFN-γ^+^TNF-α^+^) SL8-specific CD8^+^ T cells were present in the spleens of immunized mice relative to nonimmunized mice (Fig. 6B). Moreover, greater numbers of monofunctional and polyfunctional SL8-specific CD8^+^ T cells were present in the spleens of mice immunized with ΔgL-SL8-MCMV plus rIL-33 relative to mice immunized with ΔgL-SL8-MCMV plus PBS (Fig. 6A, 6B). Vaccine-induced SL8-specific CD8^+^ T cells responding to rVV-OVA challenge displayed lower expression levels of CD27 and PD-1 and higher expression levels of KLRG-1 compared with de novo SL8-specific CD8^+^ T cells responding to rVV-OVA challenge (Fig. 6C). These phenotypic trends were further emphasized in mice immunized with ΔgL-SL8-MCMV plus rIL-33 (Fig. 6C). Greater numbers of SL8-specific CD8^+^ T cells with a CD27^lo^KLRG-1^hi^ memory phenotype were therefore present in the spleens of mice immunized with ΔgL-SL8-MCMV plus rIL-33 relative to mice immunized with ΔgL-SL8-MCMV plus PBS (Fig. 6D, 6E). Such effector-like cells have previously been associated with protection against pathogenic challenge (70).

Systemic challenge with recombinant VV leads to viral replication in the ovaries, and immune protection is conferred by Ag-specific CD8^+^ T cells (69). Accordingly, we used a plaque assay to quantify replicating rVV-OVA in the ovaries of naive and immunized mice. Vaccination substantially reduced virus load after challenge with rVV-OVA (Fig. 6F). Moreover, rIL-33 further enhanced immune control, such that lower virus loads were observed in mice immunized with ΔgL-SL8-MCMV plus IL-33 relative to mice immunized with ΔgL-SL8-MCMV plus PBS (Fig. 6F).

Collectively, these data show that rIL-33 can augment the immunogenicity and efficacy of a ΔgL-MCMV–based vaccine designed to elicit CD8^+^ memory T cells with antiviral effector functions that protect against subsequent heterologous challenge.

### Endogenous IL-33 promotes anamnestic MCMV-specific CD8^+^ memory T cell responses

**FIGURE 7.**
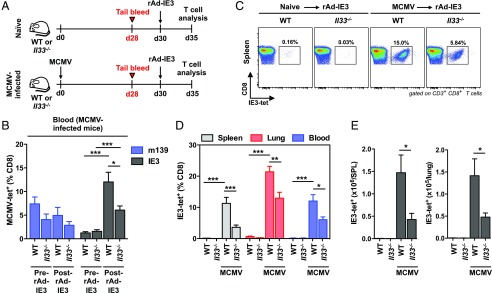
*Il33^−/−^* mice exhibit impaired recall of MCMV-specific CD8^+^ memory T cells during chronic MCMV infection. C57BL/6 (WT) and *Il33^−/−^* mice were infected with MCMV. (**A**) Schematic representation of the experimental protocol. Peripheral blood was drawn on day 28 postinfection (pi). Mice were challenged with rAd-IE3 on day 30 pi. Spleens, lungs, and peripheral blood were harvested on day 35 pi. Naive mice were challenged in parallel as controls. (**B**) Frequencies of tetramer-binding CD8^+^ T cells specific for m139 or IE3 were quantified in peripheral blood on day 28 pi and day 5 postchallenge (day 35 pi). Data are shown as mean ± SEM (*n* = 4 mice per group). (**C**) Frequencies of tetramer-binding CD8^+^ T cells specific for IE3 were quantified among leukocytes isolated from spleens on day 5 postchallenge (day 35 pi). Representative flow cytometry plots are shown. (**D**) Frequencies of tetramer-binding CD8^+^ T cells specific for IE3 were quantified among leukocytes isolated from spleens, lungs, and peripheral blood on day 5 postchallenge (day 35 pi). Data are shown as mean ± SEM (*n* = 4 mice per group). (**E**) Total numbers of tetramer-binding CD8^+^ T cells specific for IE3 were quantified among leukocytes isolated from spleens and lungs on day 5 postchallenge (day 35 pi). Data are shown as mean ± SEM (*n* = 4 mice per group). Results are concatenated from two independent experiments (B, D, and E). *p ≤ 0.05, **p ≤ 0.01, ***p ≤ 0.001.

Memory recall is a key determinant of effective local and systemic immunity (59). Accordingly, we tested the hypothesis that IL-33 can enhance virus-specific CD8^+^ memory T cell responsiveness to secondary Ag encounter, based on our evaluation of mice immunized with ΔgL-SL8-MCMV. For this purpose, we infected WT and *Il33^−/−^* mice with MCMV and measured recall responses triggered by rAd-IE3 (Fig. 7A). To ensure comparability across strains, challenge experiments were performed on day 30 postinfection, a time point at which equivalent frequencies of circulating IE3-specific CD8^+^ T cells were present in WT and *Il33^−/−^* mice (Fig. 7B). Low frequencies (Fig. 7C, 7D) and numbers (Fig. 7E) of IE3-specific CD8^+^ T cells were detected in the spleens, lungs, and peripheral blood of naive WT and *Il33^−/−^* mice after challenge with rAd-IE3. In contrast, substantially higher frequencies (Fig. 7B–D) and numbers (Fig. 7E) of IE3-specific CD8^+^ T cells were present in the spleens, lungs, and peripheral blood of MCMV-infected WT mice after challenge with rAd-IE3. No such differences were observed with respect to the frequencies of m139-specific CD8^+^ T cells (Fig. 7B). In addition, memory recall was dramatically impaired in the absence of endogenous IL-33, consistent with our initial predictions, such that lower frequencies (Fig. 7B–D) and numbers (Fig. 7E) of IE3-specific CD8^+^ T cells were present in the spleens, lungs, and peripheral blood of *Il33*^−/−^ mice relative to WT mice after challenge with rAd-IE3.

Collectively, these data show that IL-33 can promote anamnestic CD8^+^ memory T cell responses during chronic MCMV infection, illustrated in the context of heterologous challenge with a single epitope derived from IE3.

## Discussion

In this study, we investigated the immunomodulatory effects of IL-33 in mice infected with MCMV. The key findings were: 1) endogenous IL-33 promotes virus-specific CD8^+^ memory T cell inflation and the accumulation of CD69^+^ T_RM_ cells; 2) exogenous IL-33 further enhances virus-specific CD8^+^ memory T cell inflation and the accumulation of CD69^+^ T_RM_ cells; 3) exogenous IL-33 augments the immunogenicity and efficacy of a model vaccine based on a replication-deficient strain of MCMV; and 4) endogenous IL-33 promotes anamnestic virus-specific CD8^+^ memory T cell responses. These data identify IL-33 as an important mediator of antiviral T cell immunity and suggest that alarmins may find utility as adjuvants in the context of emerging vaccines based on attenuated derivatives of CMV.

IL-33 provides a danger signal that alerts the immune system to viral attack (31, 32, 50, 51, 53, 71). In agreement with previous studies (50, 51), we found that IL-33 was produced in the spleen and inguinal LNs in response to systemic infection with MCMV. Moreover, active viral replication was required to induce IL-33. Despite this innate response, exogenous IL-33 administered at the time of virus inoculation further enhanced memory T cell inflation and the induction of CD69^+^ T_RM_ cells during chronic infection, raising the possibility of an unknown viral escape mechanism designed to subvert T cell immunity (72). However, exogenous IL-33 induced substantial weight loss in mice infected with replication-competent MCMV. These observations suggest that immune enhancement may be counteracted in excess by the proinflammatory properties of IL-33.

In contrast to a recent study of *St2^−/−^* BALB/c mice (51), we found that IL-33 was not required for the induction of virus-specific CD8^+^ T cell responses during acute MCMV infection. Our data nonetheless concur with the observation that immune control of viral replication was not impacted by the lack of ST2 (51). We also found that Ly49H^+^ NK cells accumulated in the spleen irrespective of IL-33, again in apparent disagreement with earlier studies using different models (50). In contrast, protective CD8^+^ T cells are critically dependent on IL-33 and ST2 during acute infection with LCMV or VV (31). It is notable in this context that CD4^+^ T cell help is not required for the induction of virus-specific CD8^+^ T cell responses to LCMV or MCMV (73–75). However, these viruses differ markedly with respect to Ag presentation, which in turn may impact the sensitivity of immune priming events to IL-33. In particular, MCMV-specific CD8^+^ T cells are primed by DCs capable of cross-presentation, such as lymphoid-resident CD8α^+^ cDCs (76, 77). Our data further show that CD8α^+^ cDCs are not depleted in *Il33^−/−^* mice. In contrast, LCMV-specific CD8^+^ T cells can be primed by APCs other than DCs (78), such as macrophages (79) and nonhematopoietic cells (80).

A different picture emerged from the dataset acquired during chronic MCMV infection. In this setting, lower frequencies and numbers of virus-specific CD8^+^ T cells were detected in the lymphoid organs and mucosal tissues of *Il33^−/−^* mice relative to WT mice. Moreover, a single dose of rIL-33 administered at the time of infection further enhanced CD8^+^ memory T cell inflation. These phenomena most evidently affected IE3-specific CD8^+^ memory T cells, which are particularly dependent on CD4^+^ T cell help (73). Although the causative mechanisms remain unknown, we noted that MCMV-specific CD8^+^ T cells, especially those with a central memory (T_CM_) phenotype, accumulated to a greater extent in the peripheral LNs of mice treated with rIL-33. This observation is pertinent in light of the prevailing view that memory inflation is driven by replenishment from a pool of virus-specific T_CM_ cells exposed to continual stimulation from infected nonhematopoietic cells in the peripheral lymphoid compartment (81, 82).

Virus-specific CD4^+^ and CD8^+^ T_RM_ cells are recruited to mucosal tissues during MCMV infection (8–10). Earlier work further identified IL-33 as an important driver of the T_RM_ phenotype (9, 60, 64), alongside TGF-β (8, 83–85). In line with these observations, we found lower frequencies and total numbers of CD8^+^ T_RM_ (CD69^+^CD103^–^ or CD69^+^CD103^+^) cells in the salivary glands of *Il33^−/−^* mice relative to WT mice. The frequencies of inflationary MCMV-specific CD8^+^ T_RM_ cells with a CD69^+^CD103^+^ phenotype, especially those specific for IE3, were also reduced in the salivary glands of *Il33^−/−^* mice relative to WT mice. However, these effects did not extend to tissue-localized CD69^–^CD103^–^ and CD69^–^CD103^+^ CD8^+^ T cells, which were retained at comparable frequencies in both strains of mice. Of note, TGF-β does not influence the expression of CD69 on T_RM_ cells (9, 60), but it can upregulate CD103 (8), which is nonetheless dispensible for tissue-resident immunity against MCMV (8, 9). In parallel evaluations, we found that the accumulation of CD4^+^ T_RM_ (CD11a^+^CD69^+^) cells was similarly impaired in the salivary glands of *Il33^−/−^* mice relative to WT mice. Overall, these data are consistent with the notion that IL-33 positively regulates the generation of CD69^+^ T_RM_ cells in response to systemic infection with MCMV.

Attenuated derivatives of CMV have shown promise as novel vaccine vectors in model systems (25). However, clinical development will likely be hampered by key limitations, most notably a relative lack of immunogenicity compared with replication-competent strains of CMV. To overcome this potential bottleneck, we investigated the immunomodulatory effects of rIL-33, which has been used previously as a vaccine adjuvant (31, 34–36). In line with the observation that viral replication is required to induce endogenous IL-33, we found that the immunogenicity of ΔgL-SL8-MCMV was enhanced by a single dose of rIL-33. After subsequent heterologous challenge, greater numbers of SL8-specific CD8^+^ T cells were also present in the spleens, lungs, and ovaries of mice immunized with ΔgL-SL8-MCMV plus rIL-33 relative to mice immunized with ΔgL-SL8-MCMV plus PBS. These adjuvant effects were associated with better control of rVV-OVA. In a further series of experiments controlled for variable inflation, we found that endogenous IL-33 was required for optimal virus-specific CD8^+^ memory T cell responses to secondary Ag exposure, delivered in the form of rAd-IE3. Moreover, rIL-33 enhanced CD4^+^ memory T cell formation in response to ΔgL-SL8-MCMV. It is notable in this light that CD4^+^ T cell help can promote the responsiveness of MCMV-specific CD8^+^ T cells (74). Accordingly, rIL-33 may act both directly and indirectly to boost the protective efficacy of ΔgL-SL8-MCMV.

In summary, our data indicate that IL-33 is necessary for the optimal development of virus-specific T_EM_ and CD69^+^ T_RM_ cells under conditions of persistent antigenic stimulation and identify a straightforward approach that can potentiate the therapeutic benefits of recombinant immunogens vectored by replication-deficient strains of CMV. Although further studies are required to validate these findings in other systems, we anticipate that the adjuvant effects of rIL-33 may enhance the general utility of vaccines that deliver a transient immunogenic stimulus, exemplified in this study by ΔgL-SL8-MCMV.

## Supplementary Material

Data Supplement
