# Supplementary material for: IL-33 Augments Virus-Specific Memory T Cell Inflation and Potentiates the Efficacy of an Attenuated Cytomegalovirus-Based Vaccine
Source: J Immunol. 2019 Jan 13;202(3):943–55. doi: 10.4049/jimmunol.1701757 (PMC6341181; doi:10.4049/jimmunol.1701757)
Supplement: Data Supplement [file JI_1701757.zip › JI_1701757_Supplemental_Figures_1.pdf]

Supplemental Figure S1

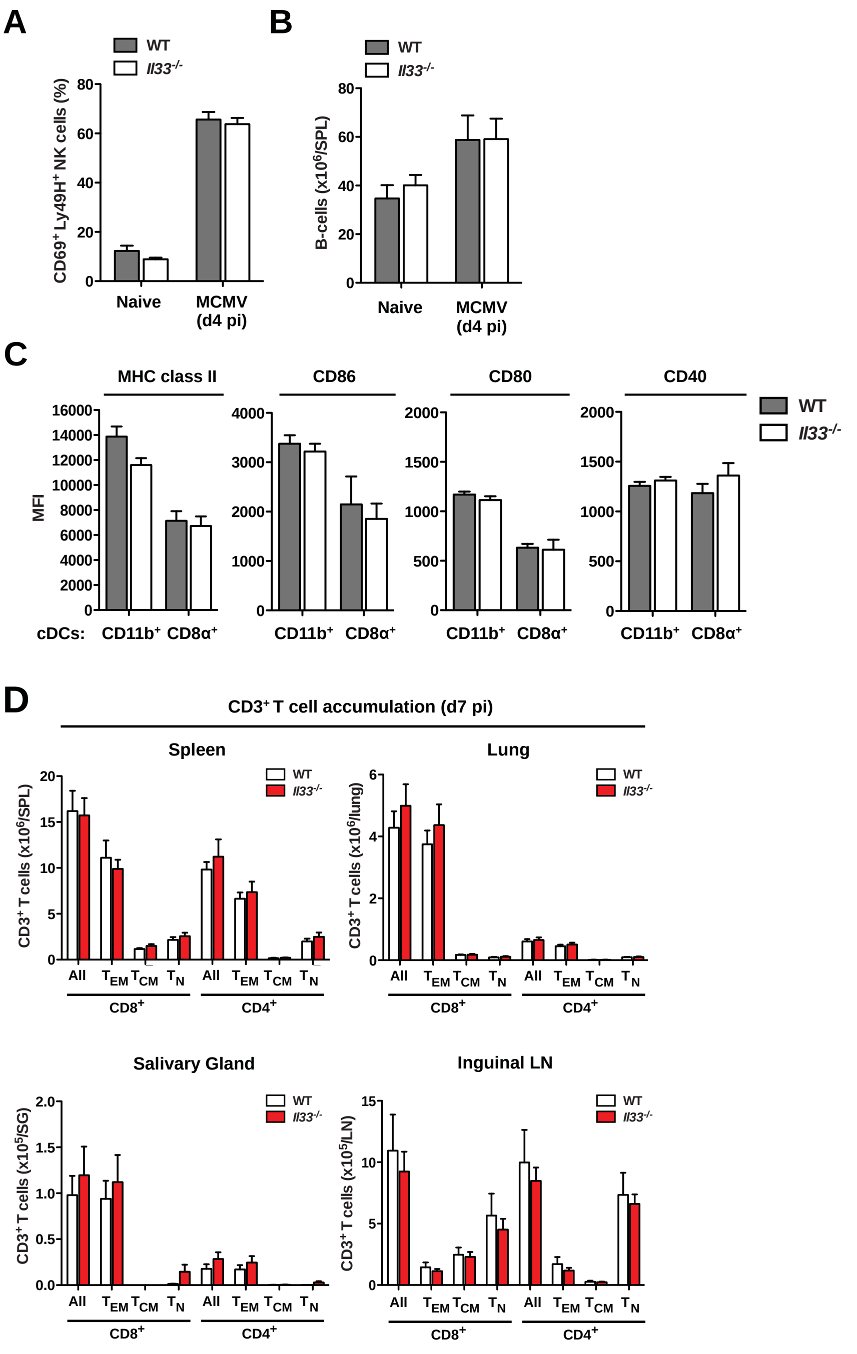

Figure S1 – IL-33 is not required for NK cell or cDC activation nor the induction of virus-specific T cell immunity during acute MCMV infection

C57BL/6 (WT) and *Il33*$^{–/–}$ mice were infected with MCMV. (**A**) Frequencies of Ly49H^{+} NK cells (CD3$^{–}$NK.1.1^{+}) expressing CD69 were quantified among splenocytes isolated on day 4 pi. Data are shown as mean ± SEM (n = 4–5 mice/group). (**B**) Total numbers of B-cells (B220^{+}Siglec-H$^{–}$) were quantified among splenocytes (SPL) isolated on day 4 pi. Data are shown as mean ± SEM (n = 4–5 mice/group). (**C**) Expression levels MHC class II and costimulatory molecules (CD40, CD80, and CD86) were quantified on CD11b^{+} (CD3$^{–}$CD11c^{+}B220$^{–}$CD8α$^{–}$CD11b^{+}) and CD8α^{+} cDCs (CD3$^{–}$CD11c^{+}B220$^{–}$CD8α^{+}CD11b$^{–}$) among splenocytes isolated on day 4 pi. Total numbers of naïve (T_N) and memory T (T_{EM} and T_{CM}) cells within the CD4^{+} and CD8^{+} compartments were quantified among leukocytes isolated from spleens (SPL), lungs, salivary glands (SG), and inguinal LNs on day 7 pi. Data are shown as mean ± SEM (n = 4–5 mice/group). MFI, mean fluorescence intensity. All results are shown (panels A–D).

Supplemental Figure S2

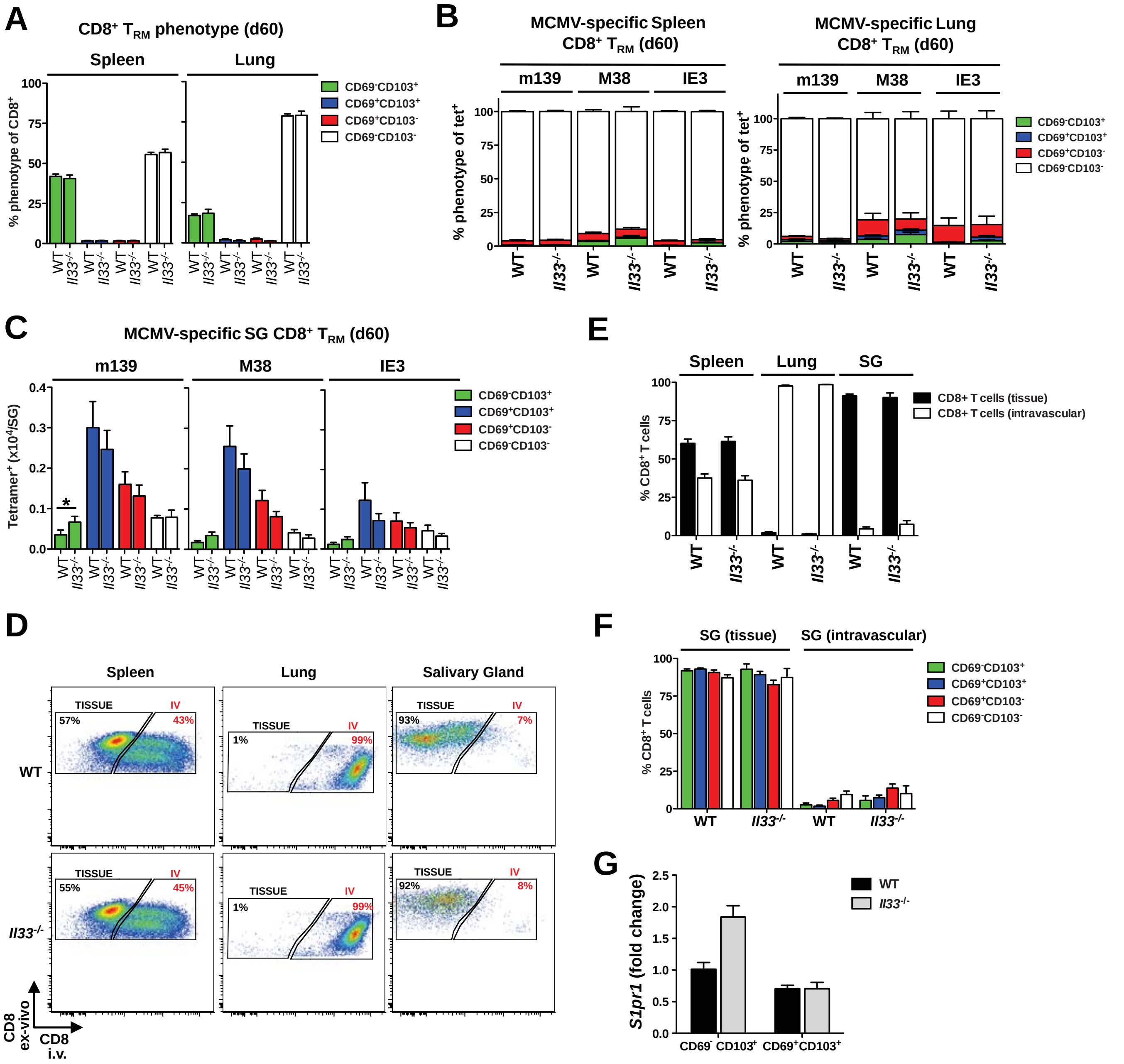

Figure S2 – IL-33 upregulates CD69 expression on virus-specific CD8⁺ T_{RM} cells

C57BL/6 (WT) and *Il33*$^{–/–}$ mice were infected with MCMV. (**A**) Expression levels of CD69 and CD103 were quantified on CD8$^+$ T cells among leukocytes isolated from spleens and lungs on day 60 post-infection (pi). Data are shown as mean ± SEM (n = 9 mice/group). (**B**) Expression levels of CD69 and CD103 were quantified on tetramer-binding CD8$^+$ T cells specific for m139, M38, or IE3 among leukocytes isolated from spleens and lungs on day 60 pi. Data are shown as mean ± SEM (n = 9 mice/group). (**C**) Total numbers of tetramer-binding CD8$^+$ T cells specific for m139, M38, or IE3 were quantified according to phenotype (CD69$^–$CD103$^+$, CD69$^+$CD103$^+$, CD69$^+$CD103$^–$, or CD69$^–$CD103$^–$) among leukocytes isolated from salivary glands (SG) on day 60 pi. Data are shown as mean ± SEM (n = 9 mice/group). Results are drawn from two independent experiments (panels A–C). (**D**) Expression levels of CD8α were quantified on viable CD3$^+$CD4$^–$ leukcoytes isolated from spleens, lungs, and salivary glands after i.v. staining on day 60 pi. Representative flow cytometry plots are shown. **(E)** Frequencies of i.v. and tissue-localized CD8$^+$ T cells were quantified among leukocytes isolated from spleens, lungs, and salivary glands on day 60 pi. Data are shown as mean ± SEM (n = 5 mice/group). **(F)** Frequencies of i.v. and tissue-localized CD8$^+$ T cells were quantified according to phenotype (CD69$^–$CD103$^+$, CD69$^+$CD103$^+$, CD69$^+$CD103$^–$, or CD69$^–$CD103$^–$) among leukocytes isolated from salivary glands on day 60 pi. Data are shown as mean ± SEM (n = 5 mice/group). (**G**) Expression of *S1pr1* was measured using a real-time quantitative PCR. Absolute values were normalized to *ACTB*. Data are shown as mean ± SEM (n = 3 mice/group). All results are shown (panels D–G).

Supplemental Figure S3

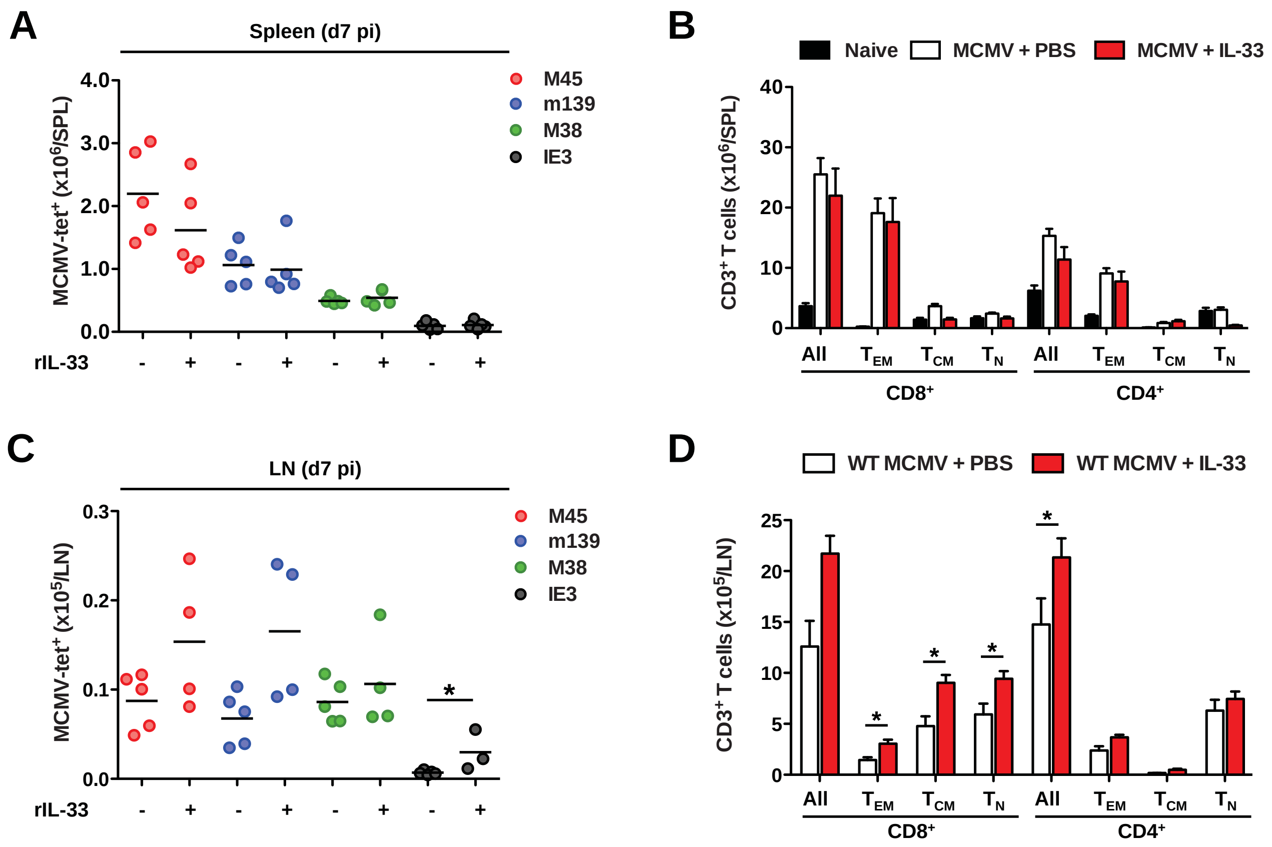

Figure S3 – rIL-33 does not influence virus-specific CD8^{+} T cell immunity during acute MCMV infection

Female C57BL/6 mice were infected with MCMV alongside a single dose of rIL-33 (2 µg) or volume-equivalent PBS. (**A**) Total numbers of tetramer-binding CD8^{+} T cells specific for M45, m139, M38, or IE3 were quantified among splenocytes (SPL) isolated on day 7 pi. Data are shown as mean ± SEM (n = 3–5 mice/group). (**B**) Total numbers of naïve (T_N) and memory T (T_{EM} and T_{CM}) cells within the CD4^{+} and CD8^{+} compartments were quantified among splenocytes (SPL) isolated on day 7 pi. Data are shown as mean ± SEM (n = 3–5 mice/group). (**C**) Total numbers of tetramer-binding CD8^{+} T cells specific for M45, m139, M38, or IE3 were quantified among leukocytes isolated from inguinal LNs on day 7 pi. Data are shown as mean ± SEM (n = 3–5 mice/group). (**D**) Total numbers of naïve (T_N) and memory T (T_{EM} and T_{CM}) cells within the CD4^{+} and CD8^{+} compartments were quantified among leukocytes isolated from inguinal LNs on day 7 pi. Data are shown as mean ± SEM (n = 3–5 mice/group). Results are drawn from two independent experiments (panels A–D).

Supplemental Figure S4

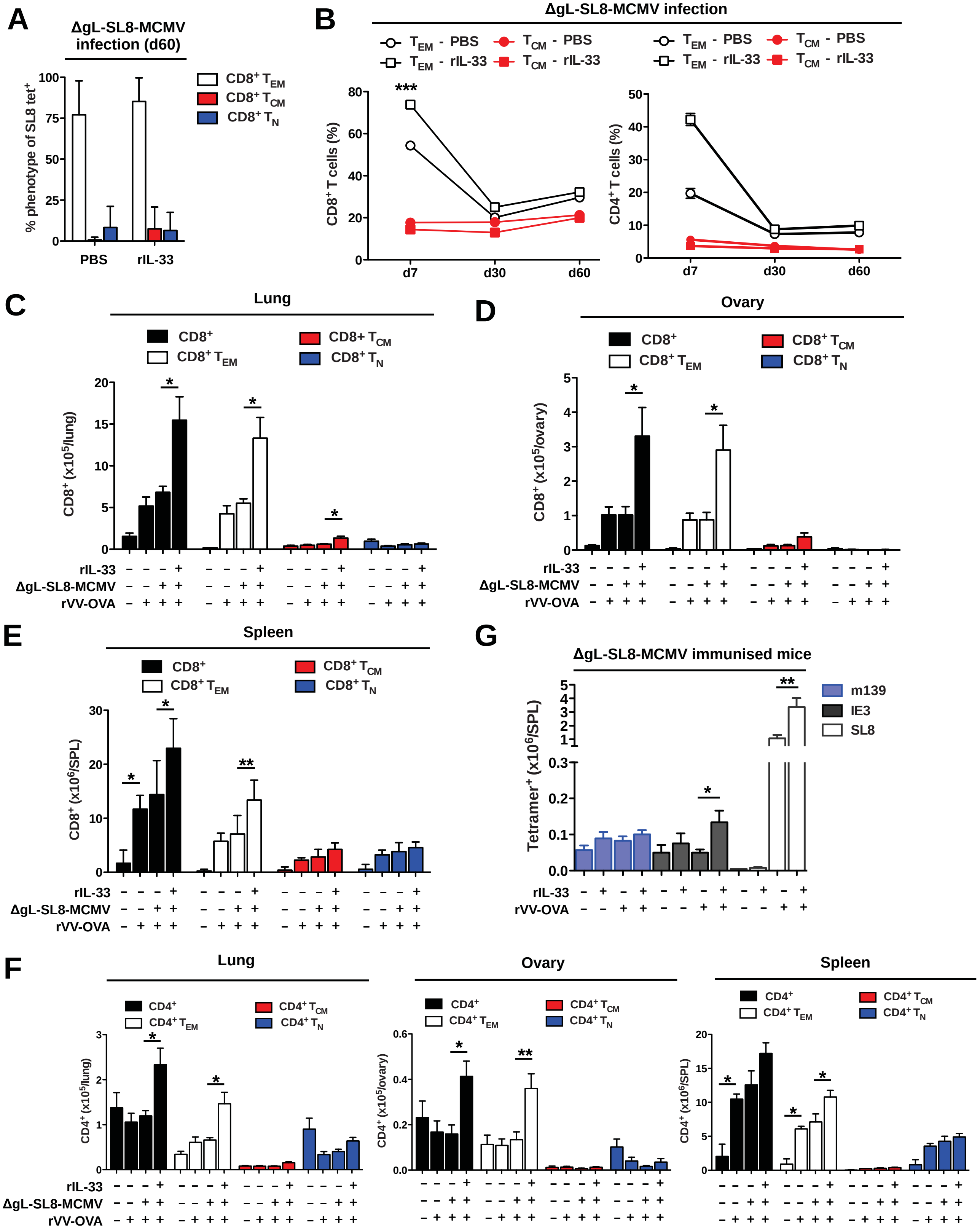

Figure S4 – rIL-33 boosts CD4⁺ and CD8⁺ T cell immunity during ΔgL-SL8-MCMV infection

Female C57BL/6 mice were infected with MCMV alongside a single dose of rIL-33 (2 µg) or volume-equivalent PBS. (**A**) Frequencies of naïve (T_N) and memory T (T_{EM} and T_{CM}) cells were quantified among tetramer-binding $CD8^+$ T cells specific for SL8 in peripheral blood on day 60 pi. Data are shown as mean ± SEM (n = 3–8 mice/group). (**B**) Frequencies of memory T (T_{EM} and T_{CM}) cells within the $CD4^+$ and $CD8^+$ compartments were quantified in peripheral blood on day 7, day 30, and day 60 pi. Data are shown as mean ± SEM (n = 3–8 mice/group). (**C**) Total numbers of naïve (T_N) and memory T (T_{EM} and T_{CM}) cells within the $CD8^+$ compartment were quantified among leukocytes isolated from lungs on day 60 pi. Data are shown as mean ± SEM (n = 3–8 mice/group). (**D**) Total numbers of naïve (T_N) and memory T (T_{EM} and T_{CM}) cells within the $CD8^+$ compartment were quantified among leukocytes isolated from ovaries on day 60 pi. Data are shown as mean ± SEM (n = 3–8 mice/group). (**E**) Total numbers of naïve (T_N) and memory T (T_{EM} and T_{CM}) cells within the $CD8^+$ compartment were quantified among splenocytes isolated on day 60 pi. Data are shown as mean ± SEM (n = 3–8 mice/group). (**F**) Total numbers of naïve (T_N) and memory T (T_{EM} and T_{CM}) cells within the $CD4^+$ compartment were quantified among leukocytes isolated from lungs, ovaries and spleen (SPL) isolated on day 60 pi. Data are shown as mean ± SEM (n = 3–8 mice/group). (**G**) Total numbers of tetramer-binding $CD8^+$ T cells specific for m139, IE3, or SL8 were quantified among splenocytes (SPL) isolated on day 60 pi. Data are shown as mean ± SEM (n = 3–8 mice/group).
